# Genetic Interaction of Centrosomin and Bazooka in Apical Domain Regulation in *Drosophila* Photoreceptor

**DOI:** 10.1371/journal.pone.0016127

**Published:** 2011-01-06

**Authors:** Geng Chen, Alicia K. Rogers, Garrett P. League, Sang-Chul Nam

**Affiliations:** Department of Biology, Baylor University, Waco, Texas, United States of America; University of Dayton, United States of America

## Abstract

**Background:**

Cell polarity genes including Crumbs (Crb) and Par complexes are essential for controlling photoreceptor morphogenesis. Among the Crb and Par complexes, Bazooka (Baz, Par-3 homolog) acts as a nodal component for other cell polarity proteins. Therefore, finding other genes interacting with Baz will help us to understand the cell polarity genes' role in photoreceptor morphogenesis.

**Methodology/Principal Findings:**

Here, we have found a genetic interaction between *baz* and *centrosomin* (*cnn*). Cnn is a core protein for centrosome which is a major microtubule-organizing center. We analyzed the effect of the *cnn* mutation on developing eyes to determine its role in photoreceptor morphogenesis. We found that Cnn is dispensable for retinal differentiation in eye imaginal discs during the larval stage. However, photoreceptors deficient in Cnn display dramatic morphogenesis defects including the mislocalization of Crumbs (Crb) and Bazooka (Baz) during mid-stage pupal eye development, suggesting that Cnn is specifically required for photoreceptor morphogenesis during pupal eye development. This role of Cnn in apical domain modulation was further supported by Cnn's gain-of-function phenotype. Cnn overexpression in photoreceptors caused the expansion of the apical Crb membrane domain, Baz and adherens junctions (AJs).

**Conclusions/Significance:**

These results strongly suggest that the interaction of Baz and Cnn is essential for apical domain and AJ modulation during photoreceptor morphogenesis, but not for the initial photoreceptor differentiation in the *Drosophila* photoreceptor.

## Introduction

Genetic control of apical-basal cell polarity is essential for epithelial morphogenesis and asymmetric cell division during cell fate specification [1]. It is also important for development of polarized subcellular structures with specialized functions such as the light sensing organelles of photoreceptor cells [2], [3]. A small number of evolutionarily conserved proteins play important roles in diverse types of apical-basal cell polarization. These polarity proteins form two major heterotrimeric cassettes consisting of Crb-Stardust (Sdt)-Patj (Crb complex) and Par-6-aPKC-Baz (Par complex) in the apical cell membrane [1]. Recent studies have shown that these two protein complexes function in a coordinated fashion [4], [5], [6], with the Par complex acting upstream of the Crb complex and Baz in the Par complex acting as the nodal point among all polarity complexes [6], [7].

**Figure 1:**
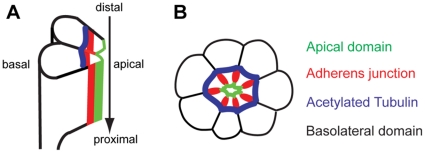
Morphogenesis of *Drosophila* pupal photoreceptors. (A) Side view of developing photoreceptors at 50% pupal development stage. The photoreceptors elongate from distal to proximal (arrow). (B) Cross-section of 50% pd pupal photoreceptors. Apical domain (green) localizes apical to AJ (red) in the center of a photoreceptor cluster. The E-cad localizes at AJ (red) which are more basal to the apical domain. The basolateral domains (black) are more basal to the AJ (red), and the acetylated-tubulin (blue) localizes at the outside from the AJs (red).

The *Drosophila* eye provides an excellent system to study *in vivo* functions of these interacting polarity proteins in control of cell polarity and organization of the rhabdomere, the light-sensitive apical structure of photoreceptor cells. In *Drosophila*, about 800 clusters of 8 photoreceptor cells (R1-R8) are generated in the eye disc epithelium during the third instar larval stage, but morphogenesis of photoreceptor cells takes place mainly during the following pupal stage [8], [9]. During early pupal stage, the apical region of each photoreceptor cell is involuted 90°, which reorients the apical side toward the center of the cluster [9]. The apical domain therefore localizes in the center and the AJ and basolateral domains surround it on all sides (Figure 1B) at the 50% pupal developmental stage. During the pupal stage, Crb complex proteins are localized immediately apical to AJs (Figure 1). During this time, developing photoreceptors undergo dramatic vertical extension from the distal region of photoreceptor cells to the proximal base of the retina.

Although it is not essential for establishing apical basal cell polarity [2], [3], Crb, together with Sdt and Patj [2], [3], [6], [10], [11], is required for the proper formation of the apical domain and AJs during photoreceptor extension along the distal-proximal axis. The mammalian homolog of Crb, CRB1, is also localized to the inner segment of photoreceptors, the structure analogous to the apical membrane domain, between the outer segment and the AJ [3]. Furthermore, mutations in *CRB1* cause retinal diseases including retinitis pigmentosa and Leber Congenital Amaurosis in human patients [12], [13].

Microtubule cytoskeletons play essential roles in determining cell shape, cell polarity, and vesicle trafficking. As a consequence, microtubule reorganization during differentiation is essential for morphogenesis. Despite their importance in cell shape and polarity generation, the organization of microtubules in *Drosophila* photoreceptors remains relatively unexplored. Therefore, we have recently examined the presence of stabilized microtubules in developing pupal photoreceptors and proposed its potential role in *Drosophila* photoreceptor development [14].

Microtubule formation takes place primarily at morphologically distinct structures termed microtubule organizing centers (MTOCs) [15]. In animal cells, centrosomes serve as the principal MTOCs. Centrosomes organize symmetric microtubule arrays of uniform polarity, where microtubule-minus ends are embedded in the centrosome while the highly dynamic-plus ends extend toward the cell periphery. In most animal cells, polarized arrays of microtubules are nucleated from the centrosome, an organelle composed of a pair of centrioles that recruit and organize a large number of proteins to form the pericentriolar matrix. Within the pericentriolar matrix, many proteins including Cnn assemble into a scaffold that docks the γ-tubulin ring complex, which nucleates and controls microtubule growth [16]. The γ-tubulin ring complex is composed of γ-tubulin and other accessory factors [17]. Cnn localizes to the pericentriolar matrix and from there other centrosomal proteins load onto the centrosome, including γ-tubulin. Centrosomes are non-functional without the addition of Cnn since it is responsible for recruitment of other centrosomal proteins. However, in many cell types microtubules are not associated with the centrosome [18]. Noncentrosomal arrays of microtubules are frequently generated in differentiated cells and are likely to expand the functional repertoire of the microtubule cytoskeleton. This is particularly true during the differentiation of specialized cell types in multicellular organisms [19]. Microtubule nucleation can also occur via centrosome-independent mechanisms. A number of microtubule-organizing structures have been identified in interphase cells. Among these are the nuclear envelope [20], plasma membrane [21], and Golgi [22], [23], [24]. Sometimes, the relocation of microtubule-anchoring proteins to noncentrosomal sites, such as the apical cell surface, occurs during development [25]. We have recently identified stabilized microtubules in developing pupal photoreceptors of *Drosophila* (Figure 1), and found that they are essential components in *Drosophila* photoreceptor morphogenesis [14].

Here we analyzed the functional role of Cnn in the localization of apical Crb and Baz during photoreceptor morphogenesis. We found that *cnn* mutant photoreceptors display severely disrupted morphogenesis with dramatic defects of the apical membrane including the Crb domain and AJs during the pupal morphogenesis stage, without affecting the early eye differentiation or pattern formation. Our data suggest that Cnn is essential for apical membrane domain modulation and for the proper morphogenesis of the developing photoreceptors.

## Results

### Genetic interaction between *baz* and *cnn* in *Drosophila* photoreceptors

**Figure 2:**
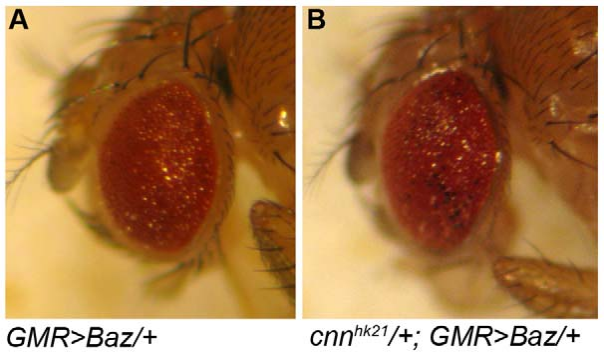
Genetic interaction of *baz* and *cnn* in *Drosophila eye*. (A–B) Adult eye phenotype of +/+; *GMR>Baz*/+ (A) and *cnn^hk21^/+*; *GMR>Baz/+* (B).

Previous studies have shown that Baz is essential for localization of Crb and AJ during photoreceptor morphogenesis [6], [7]. Furthermore, overexpressed Baz in differentiating retinal cells recruits the Crb and AJ to ectopic positions, and causes cell polarity disruptions in photoreceptors [7]. We overexpressed Baz [26] using *GMR-Gal4* [27], which led to a roughening of the eye's external morphology (Figure 2A). Using this genetically sensitized condition, we performed a genetic screen to identify additional players that function with Baz to regulate photoreceptor morphogenesis. From a pilot screen, we found that the rough eye phenotype of *GMR>Baz* was dominantly enhanced by reducing the level of *cnn* (Figure 2B). This data suggests that there is a strong genetic interaction between *baz* and *cnn*. Overexpression of Baz by *GMR-Gal4* in developing eyes resulted in severe defects in the apical basal pattern of photoreceptors, with abnormal positioning of Crb and AJ [7]. In the *cnn/+* heterozygous background to reduce the Cnn level, the rough-eye phenotype of *GMR>Baz* was strongly enhanced. This genetic interaction data strongly suggests that Cnn may provide an additional positional and/or maintenance cue for Crb and AJ positioning, because the Crb/AJ mislocalization caused by Baz overexpression further enhanced by the reduced *cnn* gene dosage (*cnn/+*) (Figure 2).

### Localization of Cnn in *Drosophila* pupal photoreceptors

**Figure 3:**
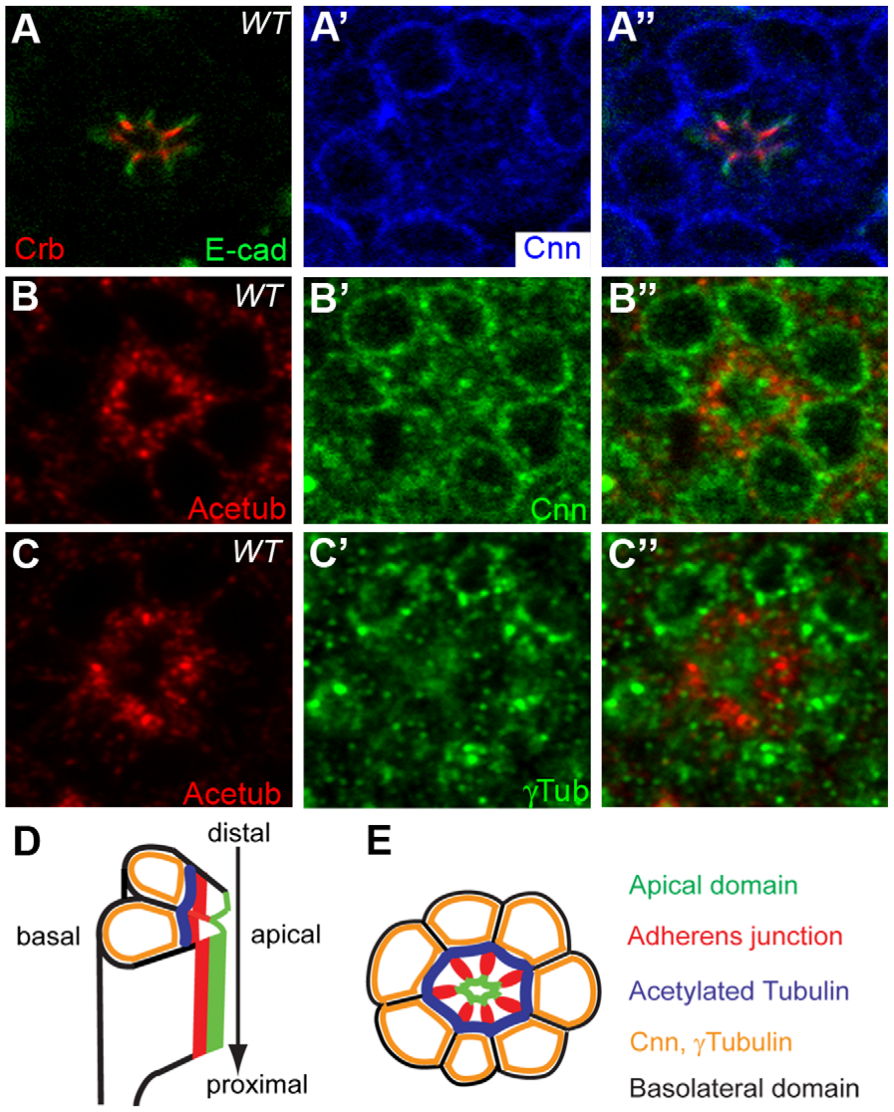
Localization of Cnn in *Drosophila* pupal photoreceptors. Localization of Cnn in mid-stage (50% pd) of pupal eyes were examined. (A) Pupal eyes were stained with Crb (apical marker, red, A), E-cad (green, AJ, A) and Cnn (blue, A′). The Cnn (blue, A′) localized more basal to the apical domain of Crb and AJ of E-cad. (B) The Cnn (green, B′) localized more basal and adjacent to the acetylated microtubules (Acetub, red, B). (C) Acetylated microtubules (red, C) localized more apical and adjacent to the γ-Tubulin (γ-Tub, green, C′).

Based on the genetic interaction between *baz* and *cnn* in the eyes, we needed to determine the localization of Cnn in developing pupal eyes, where the cell polarity genes' roles were well characterized [2], [3], [6], [7], [11]. Determining the localization of Cnn will provide information on how the acetylated microtubules are originated in developing photoreceptors and how Cnn is linked to the cell polarity genes, including *baz*. Subcellular localization of Cnn was examined at the mid-pupal stages of developing eyes by immunostaining and confocal microscopy. Cnn is highly enriched at the basal side from the apical (Crb) domain and AJ (E-cad) (Figure 3A′), and also basal and adjacent to the acetylated-microtubules (Figure 3B′) [14] in the mid-pupal eyes. The localization of Cnn was compared to that of γ-tubulin, a marker for centrosomal and non-centrosomal MTOC. Centrosome-independent MTOC has been reported [28], but the acentriolar MTOC also contains γ-tubulin and requires Cnn for its architecture and function [28]. Therefore, we examined the localization of γ-tubulin and Cnn [29] in the pupal eyes. Cnn also localizes at the same place with γ-Tubulin (Figures 3B′ and 3C′) which labels all the centrosomal MTOC as well as non-centrosomal MTOC. Since some Cnn and γ-Tubulin staining showed perinuclear distribution (Figures 3A′ and 3B′), we examined Cnn location with Elav, a nuclear marker [30], and Lamin, a nuclear membrane marker [31], [32], and found that Cnn localized at the perinuclear area in the mid-pupal eyes (data not shown). Based on these confocal image analyses, we identified the relative location of Cnn at the basal, adjacent side of the acetylated-microtubules and at the perinuclear side in the mid-pupal eyes (Figure 5). The localization pattern of Cnn strongly suggests that Cnn may contribute to the organization of microtubule arrays, rather than mitotic cell division, in post-mitotic and fully differentiated cells of the these pupal eyes. In summary, we precisely identified the exact and relative location of Cnn in developing pupal eyes using several markers of the apical domain, AJs, and acetylated-microtubules, as well as specific nuclear markers. Given the presence of Cnn underneath the acetylated microtubules in the *Drosophila* photoreceptors, Cnn may have some potential functions for photoreceptor development as a MTOC or microtubule modulator in the eyes.

### *cnn* is required for localization of acetyl-microtubules and Baz

**Figure 4:**
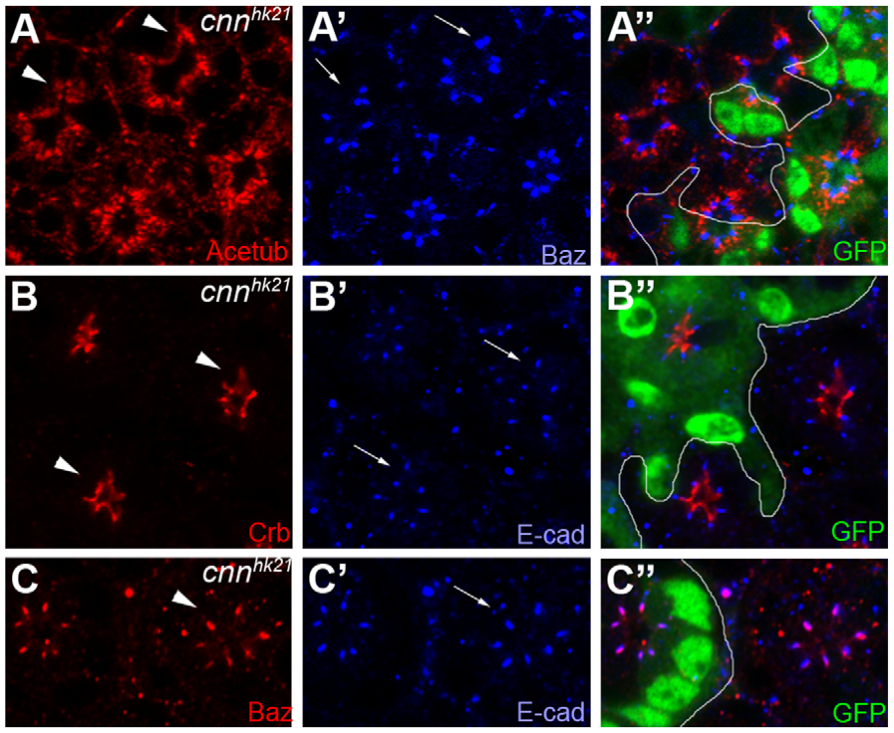
Cnn is essential for photoreceptor morphogenesis in the mid-stage developing pupal eyes. (A, B) Pupal eyes (50% pupal developmental stage) with *cnn^hk21^* null mutant clones marked by the absence of the GFP (green). (A) Acetylated tubulin (Acetub, red, A) and Baz (blue, A′) were mislocalized from apical to basal in the absence of the Cnn (absence of the GFP, A″). Ectopic mispositioned acetylated tubulins (A) and Baz (A′) are indicated by arrowheads (A) and arrows (A′), respectively. (B) Crb (red, arrowheads, B) and E-cad (blue, arrows, B′) were mislocalized from apical to basal in the absence of the Cnn (absence of the GFP, B″). (C) Baz (red, arrowhead, C) and E-cad (blue, arrow, C′) were co-mislocalized from apical to basal in the absence of the Cnn (absence of the GFP, B′).

To test whether *cnn* is required for developing photoreceptor architecture, we generated mutant clones of the *cnn* mutation using *cnn^hk21^* [29] in photoreceptors using the FLP/FRT-based genetic mosaic technique [33] with eyeless-flippase (ey-flp) [34]. Since *cnn^hk21^* is a null allele [35], we analyzed *cnn^hk21^* mutation to identify the Cnn functions. During the early larval stage, *cnn* mutants showed no defects in photoreceptor differentiation or pattern formation (data not shown). However, from the mid-pupal (50%) developmental stage, the *cnn* mutation causes the acetylated microtubules and Baz domains to mislocalize from basal to apical positions (Figure 4A). Based on statistical analysis of Baz membrane domains, about 30±5% (n = 30) of the apical domain size was increased in the *cnn* mutant (ImageJ analysis). The acetylated microtubule bundles, at the basal side of the Baz membrane domains, were also mispositioned (Figure 4A). Interestingly, the ectopic position of acetylated microtubules (Figure 4A, arrowheads) also recruited the ectopically positioned Baz (Figure 4A′, arrows) at its apical side. Crb, the apical membrane marker, also mispositioned from apical to basal in the *cnn* mutant (Figure 4B, arrowheads), which is consistent with the Baz/E-cad mispositioning in the *cnn* mutant (Figure 4A′ and 4B′). Furthermore, other apical markers (Sdt and Patj) showed the same patterns of Crb in the *cnn* mutant (data not shown). The localization of E-cad, an AJ marker, was co-distributed with Baz (Figure 4C) in the *cnn* mutant, which is consistent with the Baz localization at the AJs in the pupal eyes [6], [7]. During the extensive morphogenetic rearrangement of the photoreceptors, the absence of Cnn causes the mispositioning and/or defects of the localization of acetylated microtubules. Furthermore, this mispositioned acetylated microtubule recruits the ectopic localization of Baz which causes the mislocalization of Crb and AJ/E-cad. These mutant phenotypes of *cnn* strongly suggest that Cnn is dispensable in early eye pattern formation, but is required for the regular localizations of Crb, Baz/AJ, and acetylated tubulins during photoreceptor morphogenesis in the pupal eyes.

### Overexpression of Cnn causes apical domain expansion in pupal photoreceptors

**Figure 5:**
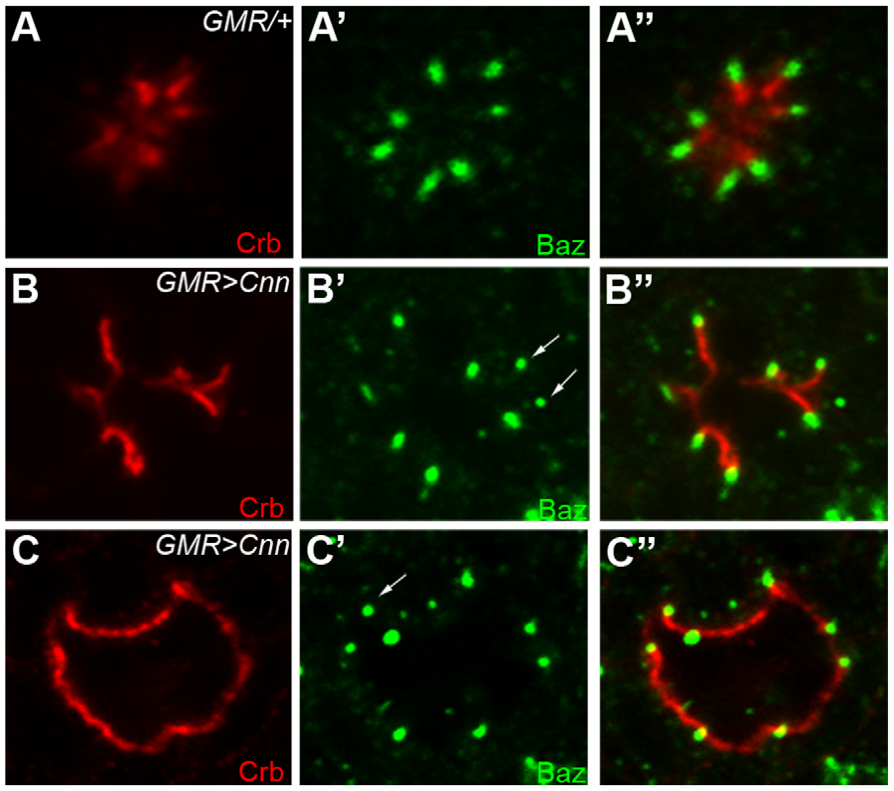
Overexpression of Cnn causes the apical domain expansions. Pupal eyes (50% pupal developmental stage) with Cnn overexpression driven by *GMR-GAL4* at 29°C were examined by Crb (green, apical domain marker), and Baz (green). (A) control, *GMR-GAL4/+*. (B, C) *GMR-GAL4/UAS-GFP-Cnn*. The expanded Crb domain (red) and Baz (green) were caused by the Cnn overexpression (B). The ectopic localizations of Baz are indicated by arrows (B′, C′).

The loss-of-function analysis of the *cnn* mutation (Figure 4) strongly suggests that *cnn* might affect AJ/Baz positioning and the apical membrane domain (Crb) in photoreceptor morphogenesis. Next we conducted a gain-of-function analysis of *cnn* using eye-specific GAL4 lines, *GMR-GAL4* [27], to increase the Cnn level in the photoreceptors. We employed the established *UAS-GFP-Cnn* [36] to examine the effects of Cnn overexpression for photoreceptor morphogenesis. Cnn overexpression in the mid-stage (50%) pupal photoreceptors dramatically expanded the apical membrane domains in an apical to basal fashion (300±100% expansion, n = 100, Figure 5B), with concurrent mispositionings of Baz (Figure 5B′) from the apical center of the photoreceptor. Similarly, the AJ marker E-cad showed the same pattern as Baz (data not shown) in the case of Cnn overexpression. In rare occasions of Cnn overexpression (<5%), the Crb domain expansion was so dramatic that the apical Crb domains were completely removed from the apical position and reallocated to a much more basal location (Figure 5C). Although the mislocalization of Crb and Baz/E-cad was dramatic, there were no defects in cell polarity since the Crb apical marker still localized more apically compared to Baz/AJ (Figure 5B and 5C). Interestingly, it was noticed that the ectopically positioned Baz always recruits Crb at its apical side (Figures 5B and 5C, arrows).

### Role of Cnn in apical domain regulation in pupal photoreceptors

Our results strongly suggest that Cnn specifically controls membrane domain size of the apical domain and AJs during pupal eye development based on the genetic interaction (Figure 2), loss-of-function (Figure 4) and gain-of-function (Figure 5) phenotypes of *cnn*. These data strongly suggest that Baz receives the primary defects created by loss of Cnn or Cnn overexpression, perhaps due to Cnn's role in microtubule organization or modulation. The apical Crb domain could be the recipient of secondary defects caused by Baz defects, since apical Crb's dependency on Baz at the AJs has been previously found in developing pupal eyes [6], [7]. However, we cannot exclude other possibilities of (1) independent control of Crb and Baz by Cnn, and (2) primary defects of Crb and subsequent secondary defects of Baz/AJs, since the apical Crb domain and Baz/AJ expansions were concurrent (Figures 4 and 5). In summary, our results strongly suggest that Cnn and its interaction with Baz are essential in photoreceptor morphogenesis.

## Discussion

We have recently demonstrated that the *Drosophila* photoreceptor cells have acetylated microtubules in their pupal stage [14]. Therefore, the microtubule modulators and organizers might have important roles in the photoreceptor development and organization. Here we examined Cnn as a potential microtubule organizer and/or modulator in the developing pupal photoreceptors. Based on our results in which (1) Cnn was identified as a genetic interaction partner of Baz, (2) Cnn localized at the basal side of the acetylated microtubules, (3) acetylated microtubules were defective in the *cnn* mutant, and (4) Baz was mislocalized in the absence of Cnn, we propose that Cnn may contribute to the organization of microtubule arrays which might in turn contribute to Baz localization, rather than mitotic cell division, in post-mitotic and fully differentiated photoreceptor cells in the pupal eyes. Therefore, the absence of Cnn causes abnormal localization of acetylated microtubules which cause the mispositioning of Baz (Figure 4A). Since Baz is essential for proper targeting of Crb and AJ [6], [7], mispositioned Baz in *cnn* mutants causes the defects of Crb and AJ (Figure 4B).

We investigated where Cnn localizes compared to other cell polarity markers, including the Crb and Par complexes, in mid-stage pupal photoreceptors. The localization results of Cnn and γ-Tubulin in pupal eyes strongly indicate that Cnn and γ-Tubulin localize at a perinuclear position, as well as adjacent to the basal side of the acetylated microtubules (Figure 3). Similar perinuclear localization of γ-Tubulin was reported in *Drosophila* myotube [37]. In the third instar larvae eye disc, it was reported that Cnn was basally positioned in epithelial neuronal precursors, based on the localization of Cnn-GFP expression [38].

Cnn has a microtubule organizing activity. Therefore, the expected results in the loss of *cnn* are the loss of acetylated microtubules in the pupal eyes. However, most of the acetylated microtubules were still present, although they were mislocalized and mispositioned from apical to basal locations in the *cnn* mutants (Figure 4A). One potential possibility is that non-centrosomal and non-Cnn-dependent-MTOC is not affected in *cnn* mutants. This possibility is supported by several reports of MTOC in the golgi and other intracellular structures [20], [21], [22], [23], [24], [25]. However, our results (Figure 4) strongly suggest that the presence of the Cnn-independent MTOCs are not sufficient to preserve the acetylated microtubules in their correct location and that Cnn is thus essential in the proper Crb/Baz localization in the photoreceptors (Figure 4).

Baz defects might not be caused by the defect of Baz membrane targeting because (1) there were no early eye defects in the larval or early pupal stage, (2) there were no cell polarity defects, and (3) only the Crb/Baz/AJ domains were defective. Therefore the *cnn* mutant defects might be the apical membrane domain controls which may be regulated by the Baz-dependent apical targeting of Par-6/aPKC or Crb/Sdt/Patj. This hypothesis is further supported by the gain-of-function of Cnn which shows a dramatic expansion of Crb (Figure 5). Further research is needed to answer these important questions.

Since Cnn functions in many diverse cellular processes, it was assumed to be impossible to examine a specific developmental defect in the photoreceptor. However, our analysis of the *cnn* mutation in the photoreceptor showed defects that are quite specific in the pupal photoreceptors based on (1) a lack of defects in early eye pattern formation during the larval stage, and (2) an absence of defects detected in eye accessory cells of pigment cells and cone cells in the pupal stage eyes (data now shown).

Differential defects in larval or pupal eye development are common, since some cell polarity genes such as *crb* are not required in larval imaginal discs (Izaddoost et al., 2002), although they become essential later during the pupal stage when the photoreceptor cells undergoes dramatic reorganization. Our analysis of pupal eyes suggests that Cnn is required for the maintenance of apical and AJ domains of photoreceptor cells, as Baz and apical markers fail to form correct localizations in *cnn* mutant clones. These pupal specific-phenotypes are similar to the defects shown previously in the eyes of Crb complex mutants [2], [3], [6]. Like *cnn* mutations, loss of these gene functions also affects the morphogenesis but not apical basal cell polarity. Since Baz is essential for proper targeting of Crb complex proteins, loss of Cnn function may result in mislocalization of Crb complex through affecting Baz localization, although it is possible that Cnn may also be directly involved in localization of Crb complex proteins independent of Baz.

We have characterized the Cnn in the developing photoreceptors and examined its roles in *Drosophila* photoreceptor morphogenesis. Understanding the genetic basis for photoreceptor organ development is important for finding cures for retinal degeneration caused by genetic defects. Recent *Drosophila* and mammalian studies suggest that the genes involved in cell polarity play important roles in the morphogenesis of photoreceptor cells. Understanding Cnn's role in Baz targeting will provide important clues in understanding the cell polarity. Because of the conservation of cell polarity genes and Cnn in higher mammals including humans, similar cooperative mechanism between Baz and Cnn could have a role in the development and degeneration of the human photoreceptor.

## Materials and Methods

### Genetics

*UAS-Baz* [26] and *GMR-Gal4* (on chromosome 3 from Andreas Bergmann, MD Anderson) were recombined on the same chromosome for a genetic modifier screening. Mitotic recombination was induced by using the FLP/FRT method for clonal analysis [33]. *cnn^hk21^* is a null allele with a nonsense mutation that truncates the protein at amino acid 106 [29]. *cnn^hk21^* mutant clones were produced by eye-specific expression of FLP in *y w ey-flp/+*; *FRT42D cnn^hk21^/FRT42D Ubi-GFP*. Overexpression of *cnn* was induced by crossing *UAS-GFP-Cnn* [36] with *GMR-GAL4* [27] at 29°C. *cnn^hk21^* and *UAS-GFP-Cnn* were obtained from Bloomington Stock Center at Indiana University.

### Immunohistochemistry

The following primary antibodies were used: mouse anti-acetylated tubulin (Sigma), 1∶1000; rabbit anti-γ-tubulin (Sigma or Abcam), 1∶1000; rabbit anti-Baz [26], 1∶1000; rat anti-E-cadherin (DSHB) [39], 1∶10; guinea pig anti-Cnn [40], 1∶1000; rat anti-Crb [41], 1∶400; rat anti-Elav (DSHB) [30], 1∶50; sheep anti-GFP (Biogenesis or Serotec), 1∶100; mouse anti-Lamin (DSHB) [32], 1∶50; mouse or rabbit anti-Patj [41], 1∶500; rabbit anti-Sdt, 1∶500 [42]. Secondary antibodies obtained from Jackson Laboratories conjugated with Cy3, Cy5, or FITC. Fluorescent immunostaining and confocal analysis of pupal eyes was carried out as reported [14], [43]. Fluorescent images were acquired on an Olympus FV1000 confocal microscope. Image analysis and quantification were performed using ImageJ and Adobe Photoshop.
